# VvGAMYB Orchestrates Multi-Signaling Networks to Modulate GA-Induced Parthenocarpic Seedless Berry Development in Grapes

**DOI:** 10.3390/ijms27198828

**Published:** 2026-10-02

**Authors:** Qiqi Wu, Ruiqiang Chao, Linjia Luo, Yuqing Huang, Jing Li, Lianshuang Liang, Xuxian Xuan, Yanan Liu, Xinpeng Chen, Xicheng Wang, Chen Wang

**Affiliations:** 1 College of Horticulture, Nanjing Agricultural University, Nanjing 211800, China; 2 Institute of Pomology, Jiangsu Academy of Agricultural Sciences, Nanjing 210014, China

**Keywords:** grape, GA, parthenocarpy, VvGAMYB, regulatory network

## Abstract

Exogenous gibberellin (GA) efficiently induces seeded grape parthenocarpy and improves berry quality, with VvGAMYB serving as a key response factor. However, the VvGAMYB-mediated regulatory networks underlying this process remain unclear. In this study, GA_3_ treatment induced ovule abortion in ‘Wink’ grape, leading to the formation of parthenocarpic seedless berries. Exogenous GA drastically repressed *VvGAMYB* levels, thereby promoting parthenocarpy in grape ovaries. Through protein interaction screening, we identified 181 predicted VvGAMYB-associated proteins, which include differentially expressed genes related to hormone signaling, sugar metabolism, and transcriptional regulation, and further revealed that GA-induced grape parthenocarpy likely operates through a VvGAMYB-mediated network, coordinating key genes such as *VvGID1B* (GA signaling), *VvRAP2-4* (ETH signaling), *VvGAPC2* (glycolysis), and *VvMETKs* (methylation modification). Among these candidates, VvGAMYB repressed *VvMETK2* promoter activity, whereas GA_3_ treatment weakened this repression, as demonstrated by a dual-luciferase assay in tobacco. Collectively, this study established an integrative framework in which VvGAMYB orchestrates phytohormone crosstalk, sugar metabolism, and epigenetic modification during GA-induced parthenocarpy. Our findings provide significant insights into the molecular network of GA-induced grape parthenocarpy and offer key target genes for the molecular breeding of high-quality seedless grapes.

## 1. Introduction

Grapes (*Vitis vinifera* L.) are cultivated worldwide, and many seeded cultivars readily undergo parthenocarpy following treatment with plant hormones, most notably gibberellin (GA) [1,2,3,4]. Numerous seeded varieties, including ‘Wink’ [5], ‘Tamnara’ [6], ‘Kyoho’, and ‘Red Globe’ [7], have been successfully induced by GA to produce seedless berries, making this a widely adopted practice in viticulture. Similarly, exogenous GA induces parthenocarpy in many other species, such as custard apple [8], apple [9,10], pear [11,12], loquat [13], and cherry [14]. Collectively, these studies highlight the central role of GA in promoting parthenocarpy.

GA exerts its physiological effects through both metabolic and signaling pathways. Genes involved in GA metabolism play key roles in parthenocarpy. For instance, silencing GA 2-oxidase (*GA2ox*) in tomato elevates GA_4_ levels and induces parthenocarpy while suppressing lateral branching [15]. Overexpression of GA 20-oxidase 1 (*GA20ox1*) or its homologs increases GA_4_ content and promotes parthenocarpic fruit set in tomato and pear [16,17]. Likewise, overexpression of GA 3-oxidase (*GA3ox*) in pea enhances active GA accumulation and induces parthenocarpy [18]. These findings indicate that elevated bioactive GA levels consistently promote parthenocarpic fruit formation.

Beyond metabolism, GA signaling transduction is central to parthenocarpy. DELLA proteins act as key negative regulators of GA signaling [19,20,21,22], integrating downstream gene networks to mediate GA’s diverse physiological functions. The number of DELLA family members varies across species: *Arabidopsis* has five [23,24,25], grapevine has three [26], and rice, barley, and tomato each have only one [27,28,29], suggesting species-specific diversity in GA signaling regulation. Functionally, silencing *SlDELLA* in tomato enhances GA biosynthesis gene expression and induces parthenocarpy [3,30,31]. The *pro^ΔGRAS^* mutation in tomato, caused by a transposon excision in the DELLA VHIID domain, results in fertilization defects and parthenocarpic fruit [32]. In *Arabidopsis*, DELLA multiple mutants exhibit a strong parthenocarpic phenotype [33]. Moreover, DELLA proteins regulate parthenocarpy by interacting with transcription factors to modulate downstream gene expression. For example, in pear, PbDELLA represses *PbCYP78A6* expression via interaction with *PbMYB56*, mediating GA_4+7_-induced parthenocarpy [34]. In grape, VvSLR1 suppresses VvmiR397a expression through interaction with VvWRKY26, promoting laccase-mediated lignin synthesis, and GA overcomes this repression to reduce lignin synthesis during parthenocarpy [4].

Downstream of DELLA proteins, GAMYB, a gibberellin-regulated MYB transcription factor, is a key transcription factor that mediates GA signaling and is involved in parthenocarpy [35,36,37]. Research on GAMYB has largely focused on its role in reproductive development. In barley, *HvGAMYB* is highly expressed in anthers, and its overexpression shortens stamens and reduces fertility [37]. In *Arabidopsis*, the *myb33/myb65* double mutant exhibits complete male sterility [38]. Loss of *OsGAMYB* function in rice leads to abnormal stamen, anther, and pollen development [39,40]. This functional conservation extends to horticultural crops. In cucumber, *CsGAMYB* rescues the *myb33/myb65* mutant phenotype, although its own overexpression causes male sterility [41]. In tomato, SlmiR159 targets *SlGAMYB* to regulate the SlmiR167/*SlARF6/8* module and promote parthenocarpy [42]. Our previous study in grape showed that GA enhances VvmiR159c-mediated cleavage of *VvGAMYB* by repressing VvSLR1, thereby promoting parthenocarpy [5]. These studies collectively underscore the importance of GAMYB in parthenocarpy.

Despite these advances, a key gap remains: how GAMYB coordinates a regulatory network that integrates multiple downstream signaling pathways, especially those involved in hormone crosstalk, sugar metabolism, and epigenetic modifications, during GA-induced parthenocarpy remains largely unknown. Current understanding is primarily limited to isolated pathways and individual genetic components, and an integrated framework is still lacking.

To address this gap, we characterized the GA-responsive protein VvGAMYB in grape and investigated the expression patterns and potential regulatory functions of the predicted VvGAMYB-associated candidates involved in hormone signaling, sugar metabolism, and epigenetic modifications during parthenocarpy. Our findings provide novel insights into the regulatory network underlying GA-induced grape parthenocarpy and seedless berry formation, establish a foundation for further exploring the role of VvGAMYB in reproductive development, and identify potential targets for breeding high-quality seedless grape varieties.

## 2. Results

### 2.1. Response of Inflorescences and Berries to GA-Induced Parthenocarpy in Seeded ‘Wink’ Grape

**Figure 1:**
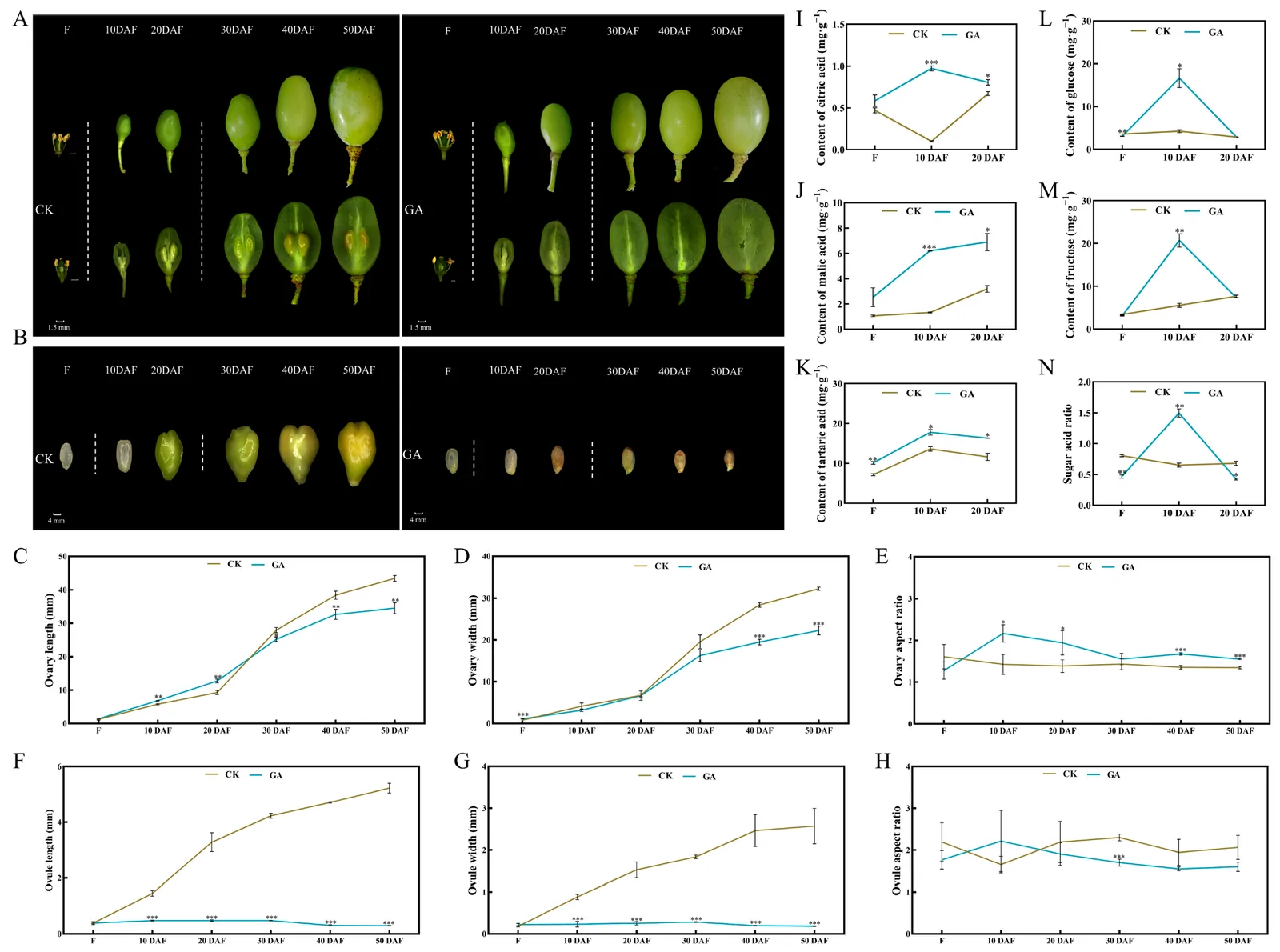
Morphological and physiological changes in ‘Wink’ grape during GA_3_-induced parthenocarpy. Morphological changes in berries (**A**) and ovules (**B**) under CK and GA_3_ treatments at different developmental stages. Changes in ovary length (**C**), ovary width (**D**), and ovary aspect ratio (**E**); ovule length (**F**), ovule width (**G**), and ovule aspect ratio (**H**); contents of citric acid (**I**), malic acid (**J**), and tartaric acid (**K**); contents of glucose (**L**) and fructose (**M**); and sugar/acid ratio (**N**). F, flowering; DAF, days after flowering. Data are presented as mean ± SD of three biological replicates. Statistical differences between CK and GA_3_ treatments at the same developmental stage were determined using a two-tailed Student’s *t*-test. *, *p* < 0.05; **, *p* < 0.01; ***, *p* < 0.001.

During GA-induced parthenocarpy in ‘Wink’ grapes, distinct alterations in reproductive organ development were observed. Specifically, from 10 to 20 days after flowering (DAF), the longitudinal growth rate of GA-treated berries transiently accelerated, reaching a length approximately 1.3-fold that of the control (CK). Thereafter, the growth rate slowed to a level comparable to that of the CK (Figure 1A). Importantly, GA treatment markedly suppressed ovule development, inducing browning, early abortion, and growth arrest as early as 10 DAF. In contrast, ovules in the CK exhibited clear morphological differentiation and continued to develop into normal seeds during the stone-hardening stage (30–50 DAF) (Figure 1B).

Further analysis revealed that GA treatment significantly elevated the peak levels of sugars and acids at 10 DAF, which reached approximately 3.8-fold and 1.7-fold of those of the CK, respectively, suggesting that sugar and acid metabolism may influence berry growth during the young fruit stage (Figure 1C–N). Collectively, these findings demonstrate that exogenous GA induces parthenocarpy in grape ovaries, accompanied by alterations in berry morphology and dynamic changes in sugar and acid metabolism, implying the involvement of a complex gene regulatory network in this process.

### 2.2. Identification and Characterization of VvGAMYB in ‘Wink’ Grape Ovaries

**Figure 2:**
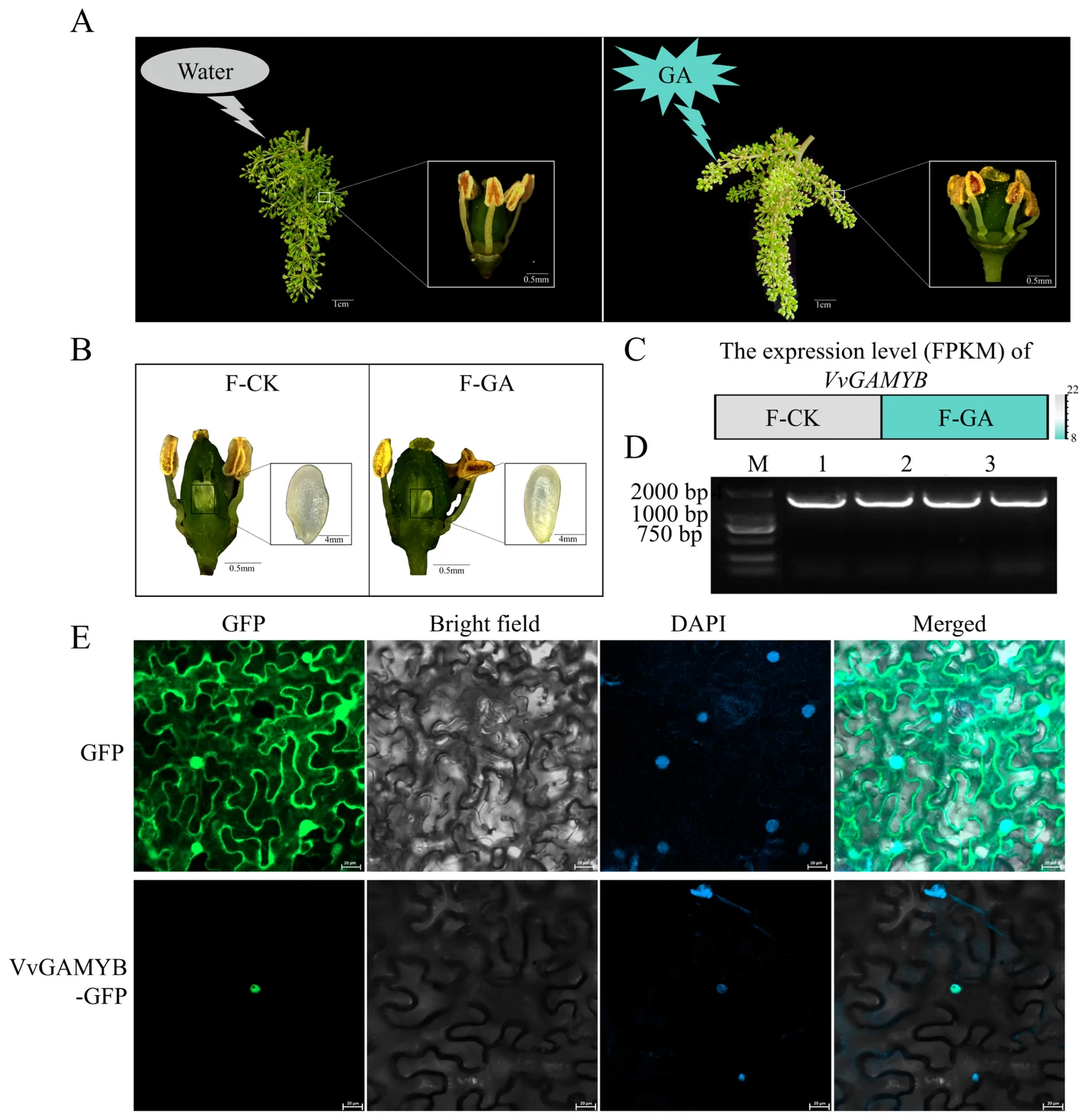
Identification and subcellular localization of the *VvGAMYB* sequence in ‘Wink’ grape. Comparison of grape inflorescence: ovary (**A**) and ovule (**B**) treated with GA in the flowering period. (**C**) RNA sequencing (RNA-seq) expression levels of *VvGAMYB* in CK and GA_3_-treated ovaries at flowering. The numerical values represent the mean values of fragments per kilobase of transcript per million mapped reads (FPKM) of three biological replicates, and the color scale directly represents mean FPKM values from low to high. (**D**) Cloning detection bands of *VvGAMYB*; ‘M’ represents a 2000 bp Marker. (**E**) Subcellular localization of heterologously expressed VvGAMYB in leaves of *N. benthamiana*. Green fluorescent protein (GFP) was used as the CK. The GFP signal was captured by laser confocal fluorescence microscopy with an emission wavelength of 488 nm. Scale bar = 20 μm.

In this study, the expression level of *VvGAMYB* was found to be significantly downregulated in ovaries during the flowering stage under GA-induced parthenocarpy in ‘Wink’ grapes (Figure 2A–C). Subsequently, *VvGAMYB* was cloned from ovule tissues of ‘Wink’ (Figure 2D), revealing a 1680 bp open reading frame (ORF) encoding a 559-amino-acid polypeptide (Figure S1A). *VvGAMYB* is located on chromosome 13, and its encoded protein was predicted to localize to the nucleus, lacking a signal peptide and transmembrane domains (Figure S1B–I). These predictions were subsequently confirmed by subcellular localization analysis in tobacco leaves, which demonstrated clear nuclear localization of VvGAMYB (Figure 2E).

**Figure 3:**
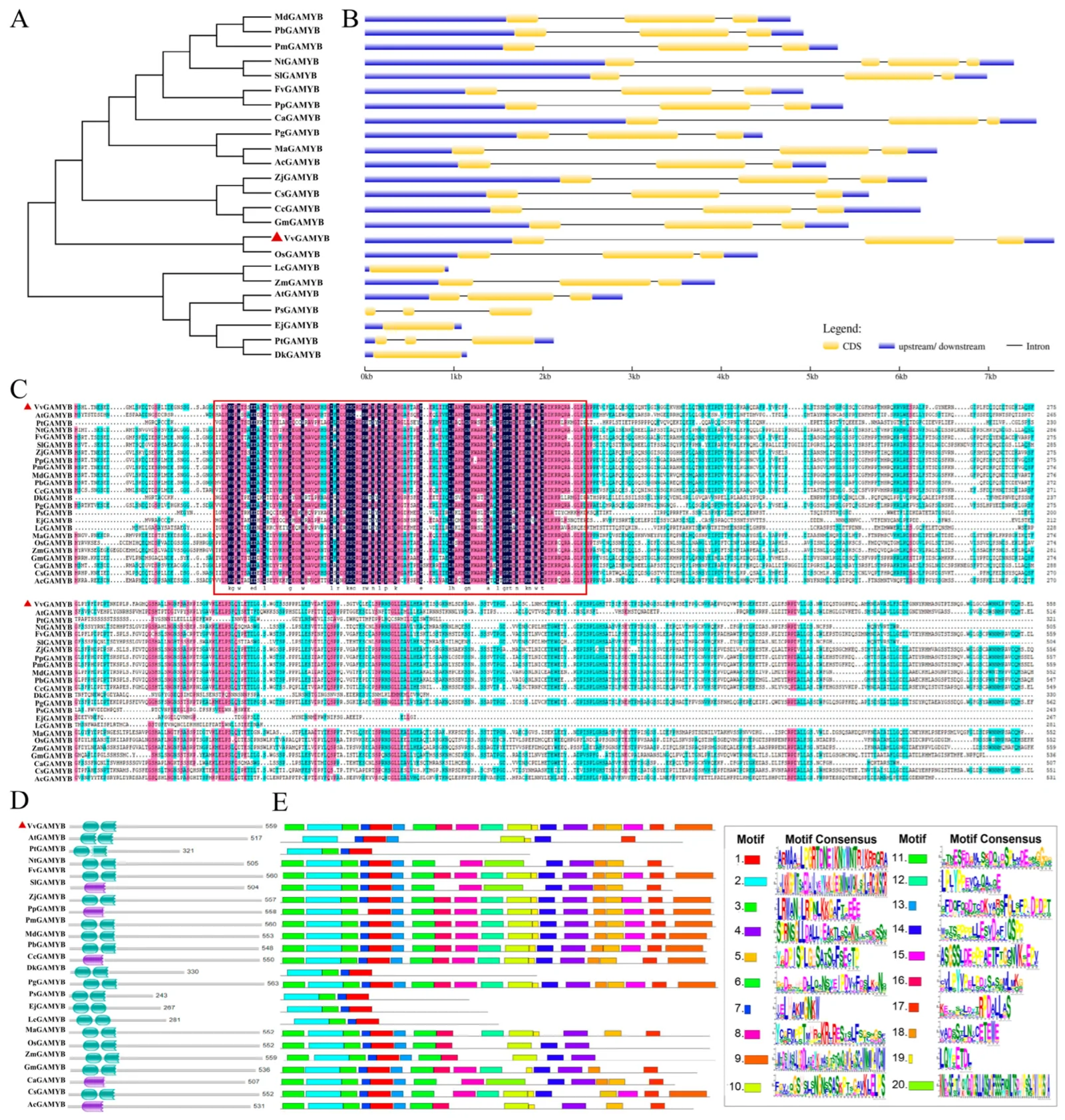
Bioinformatics analysis of homologous *GAMYB* genes across 24 plant species. (**A**) Phylogenetic tree analysis. (**B**) Gene structure distribution. (**C**) Multiple-protein-sequence alignment. (**D**) Conserved domain analysis. The red triangle indicates VvGAMYB, and the red boxes highlight the highly conserved R2R3-MYB DNA-binding region. (**E**) Motif analysis.

Subsequently, we identified homologous *GAMYB* genes across 24 plant species (Figure 3A) and revealed that *VvGAMYB* had the closest phylogenetic relationship with OsGAMYB. Most of these genes exhibited a conserved structure with one long and one short intron (Figure 3B). At the protein level, VvGAMYB and its 23 homologs contained a conserved domain at amino acid positions 50–150 (Figure 3C,D) and shared conserved motifs at equivalent positions (Figure 3E). Together, this structural conservation suggests potential functional conservation of GAMYBs across these plant species.

### 2.3. Potential-Function Analysis of Predicted VvGAMYB-Associated Proteins

#### 2.3.1. Functional Enrichment of Predicted VvGAMYB-Associated Proteins

**Figure 4:**
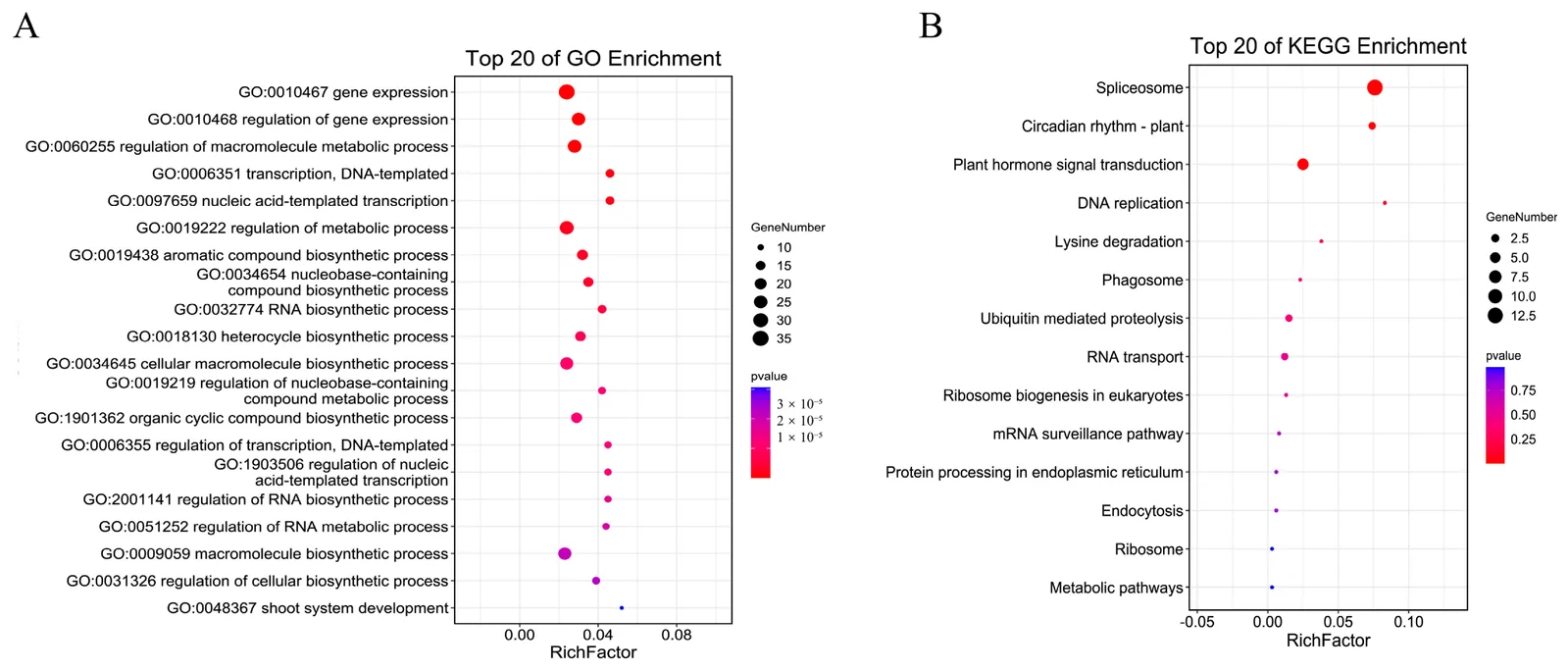
Differentially expressed genes (DEGs) among predicted *VvGAMYB*-associated candidate genes during GA-induced ‘Wink’ grape parthenocarpy. (**A**) Functional enrichment analysis of differentially expressed genes by Gene Ontology (GO) categories. (**B**) Functional enrichment analysis of differentially expressed genes in KEGG pathways. Gene members associated with each enriched GO term and KEGG pathway are listed in Table S3.

To delineate the VvGAMYB-centered regulatory networks during GA-induced parthenocarpy, we identified 181 potential VvGAMYB-associated proteins using the STRING database. Analysis of differentially expressed genes (DEGs) among these interactors revealed 90 upregulated and 91 downregulated genes (Figure S2; Table S3). Gene Ontology (GO) enrichment analysis showed that the identified DEGs were predominantly associated with biological processes, with the most significantly enriched term being “gene expression” (GO:0010467), suggesting their central role in transcriptional regulation (Figure 4A, Table S3). Further Kyoto Encyclopedia of Genes and Genomes (KEGG) pathway analysis revealed significant enrichment in pathways related to plant hormone signal transduction (ko04075) (Figure 4B, Table S3). Collectively, these results indicate that GA-induced parthenocarpy in grapes triggers transcriptional changes in predicted *VvGAMYB*-associated genes, thereby remodeling key biological networks, notably plant hormone signal transduction, and advancing our mechanistic understanding of parthenocarpy.

#### 2.3.2. Responsive Modes of *VvGAMYB* and Its Predicted Associated Genes During GA-Induced Grape Parthenocarpy

**Figure 5:**
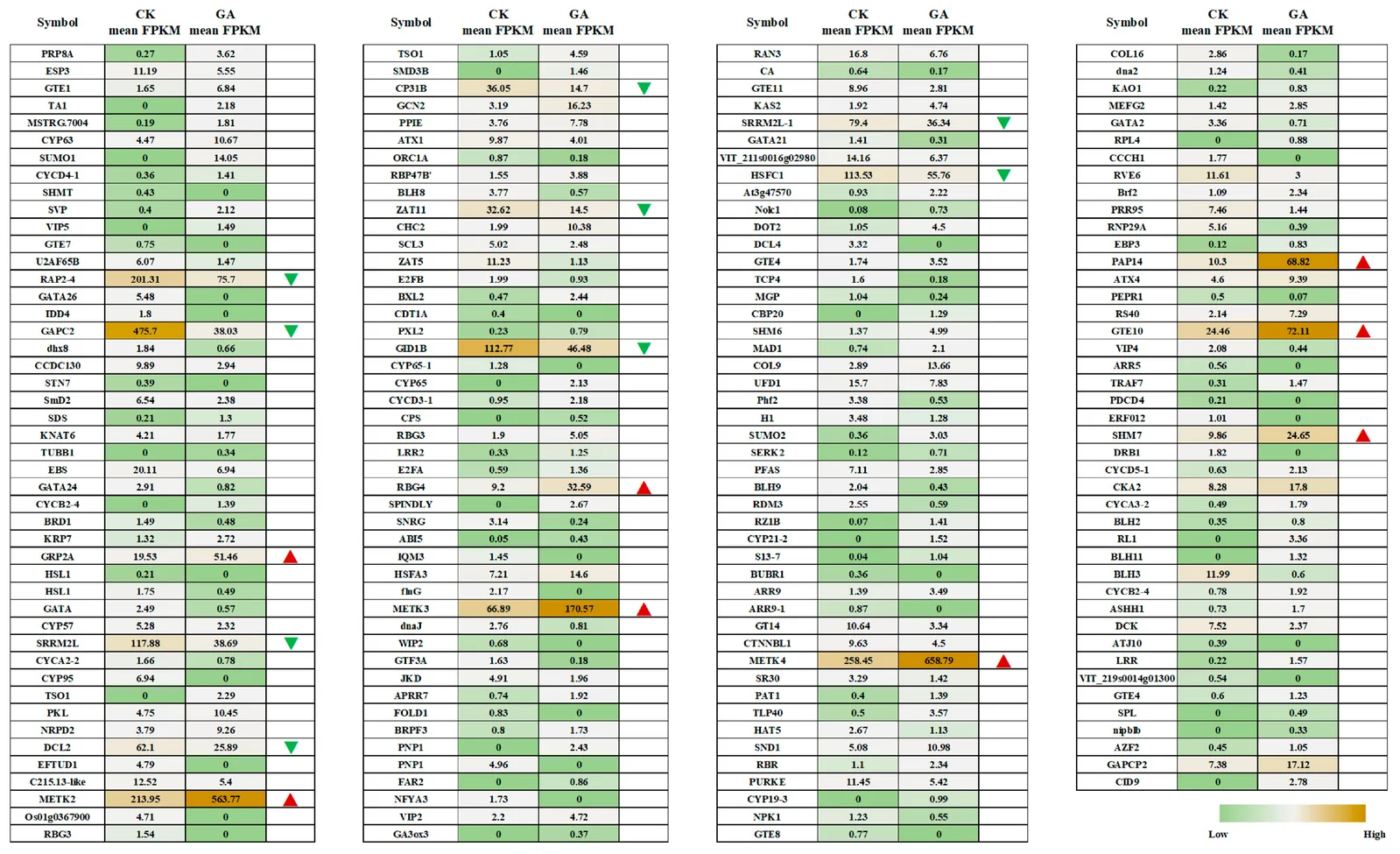
The expression profiles of 181 predicted *VvGAMYB*-associated genes under GA and CK treatments. The inverted triangle (green) represents significant downregulation after GA treatment, while the triangle (brown) represents significant upregulation.

During GA-induced grape parthenocarpy, the expression of several predicted *VvGAMYB*-associated genes was significantly upregulated, opposite to the expression pattern of *VvGAMYB* in response to GA. Specifically, VIT_216s0098g00330 (*VvPAP14*), VIT_208s0007g02860 (*VvRBG4*), VIT_217s0000g01830 (*VvGTE10*) and VIT_205s0020g00670 (*VvMETK2*) increased to 6.7-, 3.5-, 2.9-, and 2.6-fold of CK levels, respectively. In contrast, other predicted *VvGAMYB*-associated genes exhibited significant downregulation, showing an expression pattern consistent with that of *VvGAMYB*. These included VIT_201s0010g02460 (*VvGAPC2*), VIT_204s0008g02000 (*VvSRRM2L*), and VIT_200s0662g00040 (*VvRAP2-4*), which decreased to 12.5-, 3.0-, and 2.6-fold of CK levels, respectively (Figure 5; Table S2). Additionally, a subset of genes showed little to no expression changes following GA treatment, suggesting they are non-responsive to GA-induced parthenocarpy.

**Figure 6:**
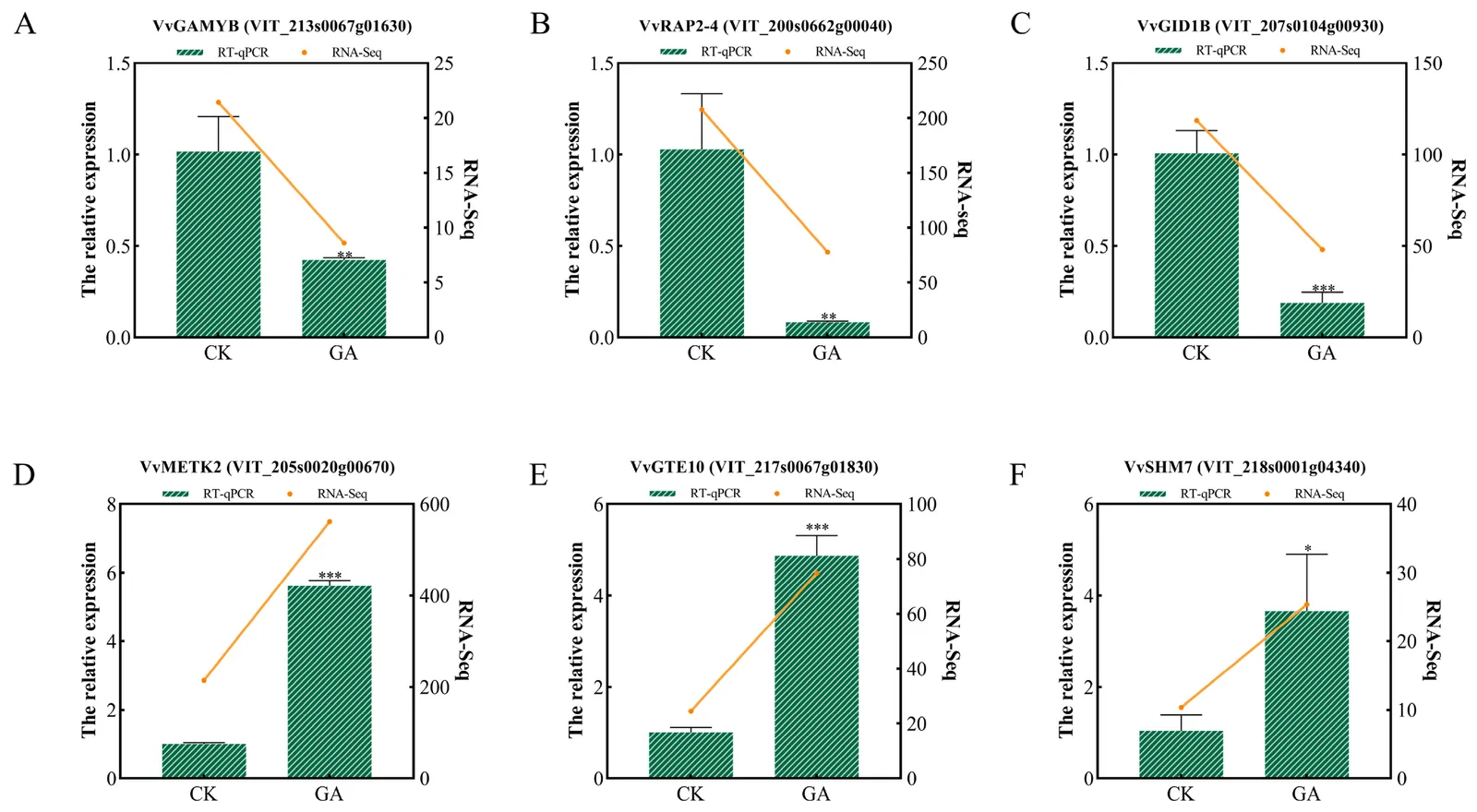
RT-qPCR validation of the RNA-seq expression patterns of selected *VvGAMYB*-associated candidate genes. (**A**) Expression pattern validation of *VvGAMYB* (**A**), *VvRAP2-4* (**B**), *VvGID1B* (**C**), *VvMETK2* (**D**), *VvGTE10* (**E**) and *VvSHM7* (**F**) under CK and GA treatments. RNA-seq data represented by FPKM values; RT-qPCR analysis normalized using the 2^−ΔΔCt^ method. Values are means ± SD from 3 independent biological replicates. Statistical significance was determined by a 2-tailed unpaired Student’s *t*-test (* *p* < 0.05; ** *p* < 0.01; *** *p* < 0.001). Error bars represent ± SD.

To validate the RNA-seq results, six genes (*VvGAMYB*, *VvRAP2-4*, *VvGID1B*, *VvMETK2*, *VvGTE10* and *VvSHM7*) were randomly selected for expression analysis by reverse transcription quantitative PCR (RT-qPCR) (Figure 6). The RT-qPCR expression profiles were highly consistent with the FPKM values derived from RNA-seq, confirming that the RNA-seq approach reliably captures the relative expression levels of these genes in GA-induced parthenocarpic grape ovaries. The reliability of the RNA-seq data was further supported by Pearson’s correlation analysis between the RT-qPCR results and FPKM values, which yielded a high coefficient of determination (*R*^2^ = 0.76) with a statistically significant *p*-value (*p* ≤ 0.01) (Figure S3).

### 2.4. VvGAMYB-Associated Regulatory Network of Multiple Signaling Pathways During GA-Induced Parthenocarpy in Grape

#### 2.4.1. Hormone Metabolism and Signaling

**Figure 7:**
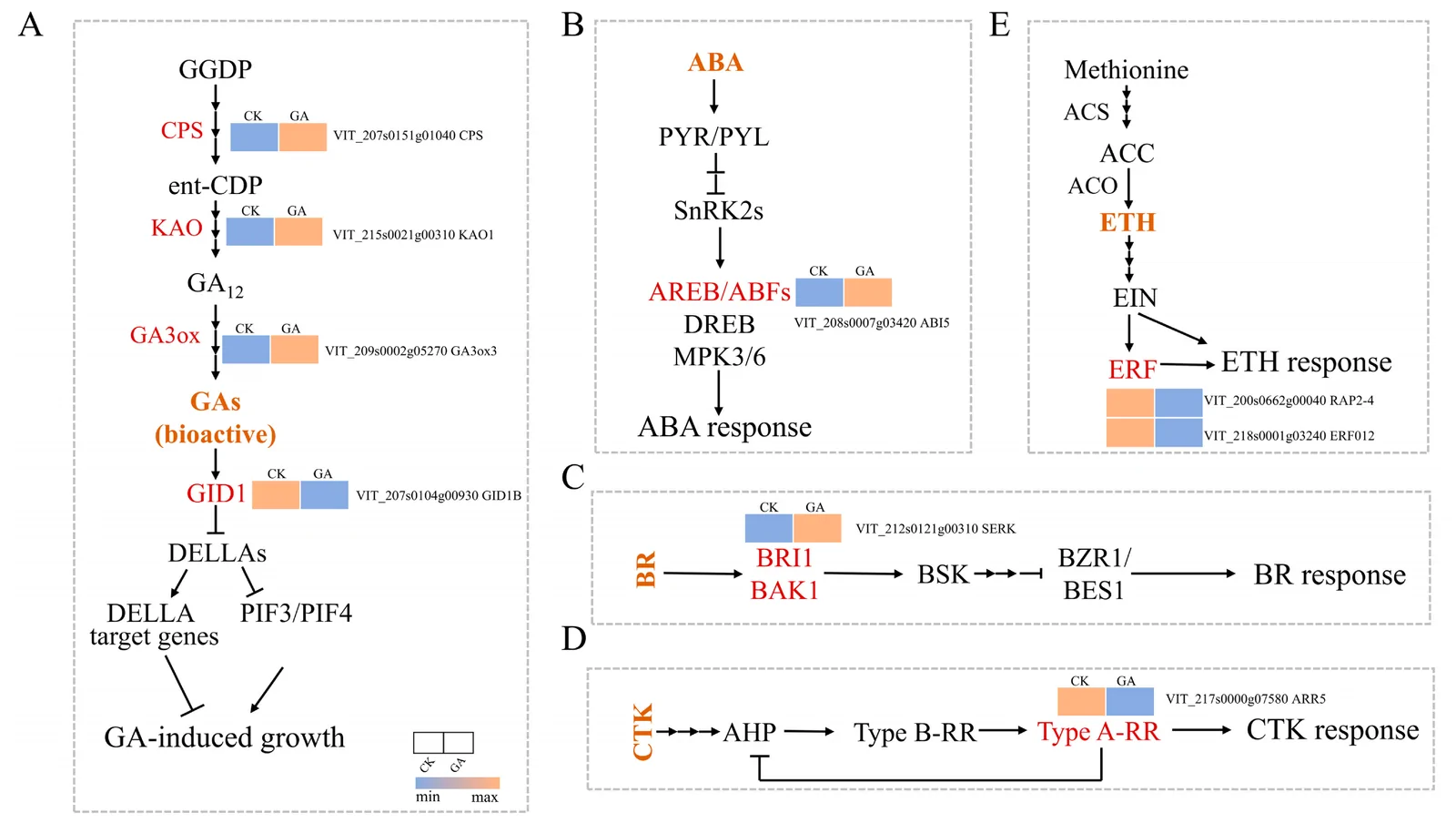
Changes in the expression of genes involved in hormone biosynthetic and signaling pathways in response to GA-induced parthenocarpy. Expression of predicted *VvGAMYB*-associated genes involved in GA (**A**), ABA (**B**), BR (**C**), CTK (**D**) and ETH (**E**) metabolism and signaling under GA conditions. The heatmap shows the relative transcript levels of selected metabolic and signaling genes for the above pathways; transcript levels are represented by mean FPKM values from three biological replicates, and colors directly represent the corresponding mean FPKM values from low to high.

Through the identification of predicted *VvGAMYB*-associated genes, we found that three genes are involved in plant hormone metabolism and six in hormone signaling, covering the GA, cytokinin (CTK), brassinosteroid (BR), abscisic acid (ABA), and ethylene (ETH) pathways. During grape parthenocarpy, exogenous GA promoted the expression of GA biosynthetic genes, including ent-copalyl diphosphate synthase (*VvCPS*), ent-kaurenoic acid oxidase (*VvKAO*), and *VvGA3ox*, which were identified among the predicted *VvGAMYB*-associated candidates. However, it inhibited the expression of the GA receptor gene *VvGID1B*, suggesting a feedback regulation mechanism in GA signaling (Figure 7A). Furthermore, exogenous GA upregulated the ABA-related gene *VvABI5* and the BR-related gene *VvSERK*, both identified as predicted *VvGAMYB*-associated candidates, while suppressing the expression of ETH signaling genes (*VvRAP2-4* and *VvERF012*) and the CTK signaling gene *VvARR5* (Figure 7B–E). Collectively, these results suggest that exogenous GA treatment may suppress *VvGAMYB* expression and promote parthenocarpy by coordinating a multi-hormone signaling regulatory network.

#### 2.4.2. Carbohydrate Metabolism

**Figure 8:**
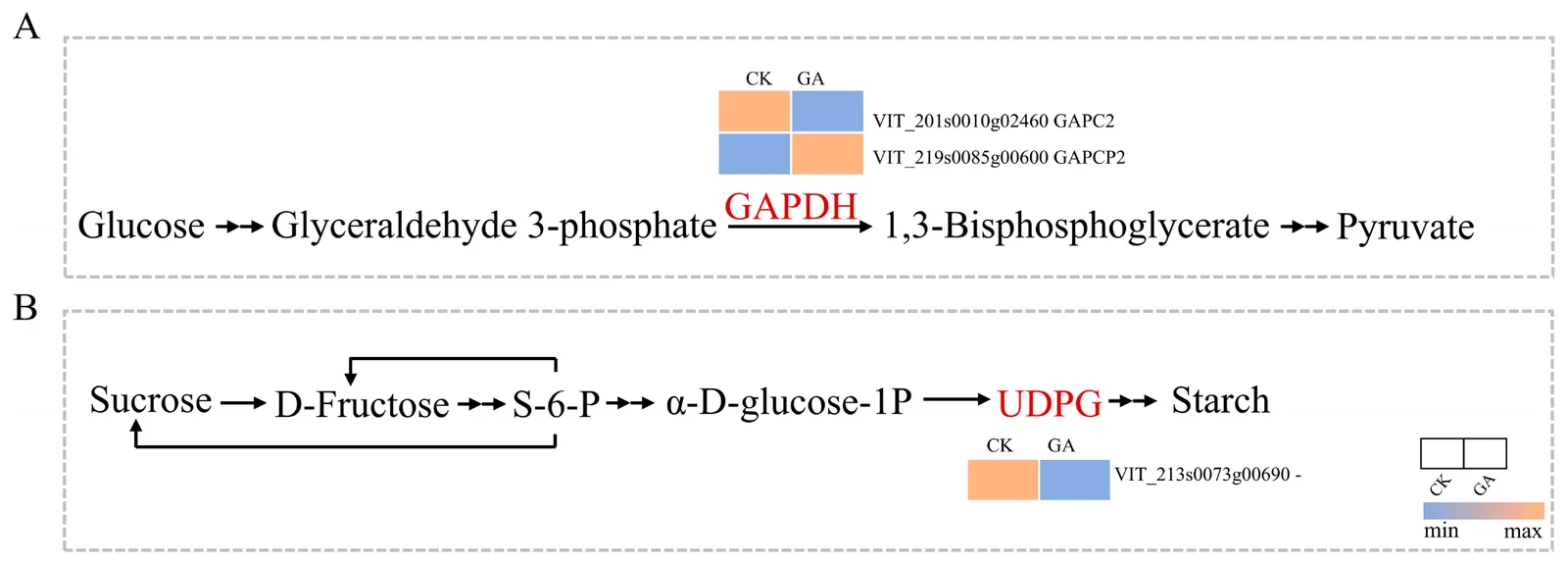
Expression patterns of *VvGAMYB*-associated candidate genes involved in glycolysis and sucrose metabolism during GA-induced parthenocarpy. (**A**) Expression of gene functions in the glycolysis pathway. GAPDH: glyceraldehyde-3-phosphate dehydrogenase. (**B**) Expression of gene function in the sucrose metabolism. S-6-P: Sucrose-6-phosphate; UDPG: UDP-glucose. Genes are grouped based on their expression patterns in GA-treated versus CK berries. And the numbers represent mean FPKM values from three biological replicates, and colors directly represent the corresponding mean FPKM values from low to high.

In GA-induced parthenocarpic grape ovaries, three genes involved in carbohydrate metabolism showed significant expression changes: two glyceraldehyde-3-phosphate dehydrogenase genes (*VvGAPC2* and *VvGAPCP2*) and one glycosyl transferase gene (VIT_219s0085g00600). Notably, the two dehydrogenase genes exhibited opposing expression patterns in response to GA. *VvGAPC2* was downregulated by 12.5-fold, mirroring the downregulation trend of *VvGAMYB* (Table S2; Figure 8A). In contrast, *VvGAPCP2* was upregulated by 2.3-fold, displaying the opposite trend to *VvGAMYB* (Figure 8A). Meanwhile, the glycosyl transferase gene was downregulated by 3.2-fold (Figure 8B), again consistent with the expression pattern of *VvGAMYB* (Table S2). The coordinated downregulation of *VvGAPC2* and the glycosyl transferase gene, alongside the counter-regulation of *VvGAPCP2*, suggests that carbohydrate metabolism is associated with the *VvGAMYB*-centered response during GA-induced parthenocarpy.

#### 2.4.3. Transcription Factors

**Figure 9:**
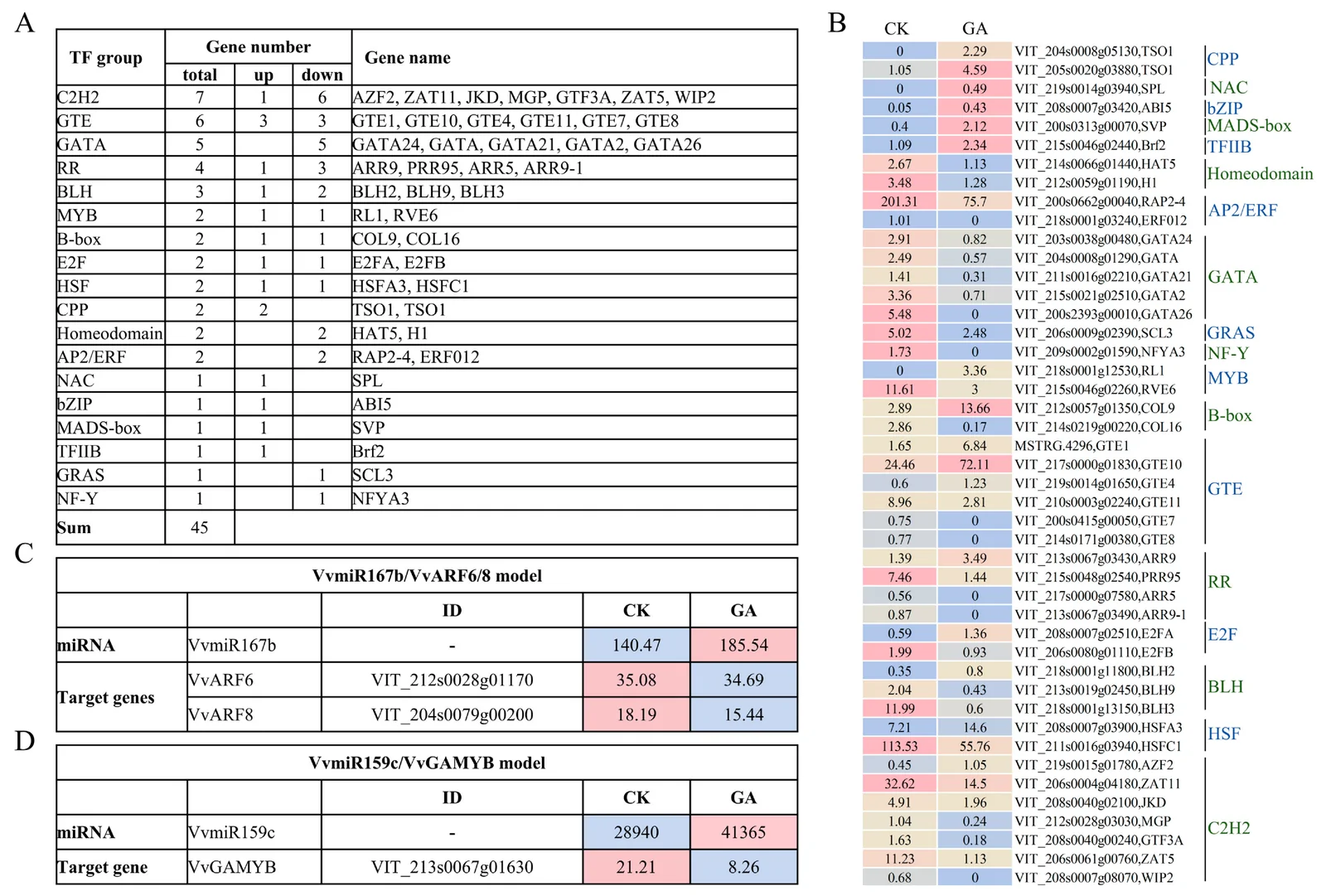
Expression levels of regulatory factors associated with the VvGAMYB-centered response during GA-induced grape parthenocarpy. (**A**) Predicted VvGAMYB-associated transcription factors showing differential expression under GA_3_ treatment. “Up” represents the number of upregulated genes after GA treatment, while “down” represents the number of downregulated genes. (**B**) RNA-seq expression profiles of 45 transcription factor genes under CK and GA_3_ treatments. Numbers within the heatmap represent mean FPKM values from three biological replicates. The color scale represents relative expression levels, with blue indicating low expression and red indicating high expression. (**C**,**D**) Proposed model for the regulatory function of miRNA/target genes under GA treatment. The heatmap shows the relative RNA-seq expression levels of regulatory genes identified. Statistical significance of the mRNA expression data was evaluated using DESeq2, and the corresponding false discovery rate (FDR) values are provided in Tables S2 and S3. FDR values for VvmiR159c and VvmiR167b were obtained from the corresponding small-RNA sequencing analysis and are provided in Table S2.

A total of 45 TFs were predicted to interact with VvGAMYB in response to GA-induced grape parthenocarpy. Based on their DNA-binding domains, these TFs were classified into 18 families, and their expression patterns are shown in a heatmap, with 16 genes upregulated and 29 downregulated (Figure 9A). The widespread downregulation of specific members from the NF-Y, GRAS, Homeodomain, AP2/ERF, and GATA families suggests that they may act in a manner similar to *VvGAMYB* (Table S2), as exemplified by the strong, 2.7-fold suppression of *VvRAP2-4*. In contrast, the upregulation of TFs from the bZIP, MADS-box, TFIIB, NAC, and CPP families indicates that they may operate in an opposing manner to *VvGAMYB* within the same regulatory network. Furthermore, eight TF families exhibited notable differential expression patterns in response to GA-induced grape parthenocarpy, where different members within the same family showed opposing trends. For instance, in the HSF family, *VvHSFA3* was upregulated by 2.02-fold, while another member, *VvHSFC1*, was downregulated by approximately 2.04-fold (Figure 9B). Collectively, this complex expression landscape depicts a multi-layered transcriptional framework that fine-tunes the downregulation of *VvGAMYB* and thereby promotes the induction of parthenocarpy in grape.

#### 2.4.4. Post-Transcriptional Regulators

MicroRNAs (miRNAs) and their target genes were also identified as participants in GA-induced grape parthenocarpy. GA treatment led to a 1.4-fold upregulation of VvmiR159c and a concurrent 2.6-fold downregulation of *VvGAMYB* (Figure 9C), suggesting that GA enhances the negative regulation of *VvGAMYB* by VvmiR159c, a finding consistent with a previous report [5]. In addition, VvmiR167b was upregulated by 1.3-fold, while its target genes *VvARF6* and *VvARF8* were downregulated by 1.0- and 1.2-fold, respectively (Figure 9D). Given that *VvGAMYB* is downregulated by GA, we speculate that this reduction may facilitate the cleavage of *VvARF6/8* transcripts mediated by VvmiR167b, thereby contributing to the regulation of parthenocarpy.

#### 2.4.5. Epigenetic Modifiers

**Figure 10:**
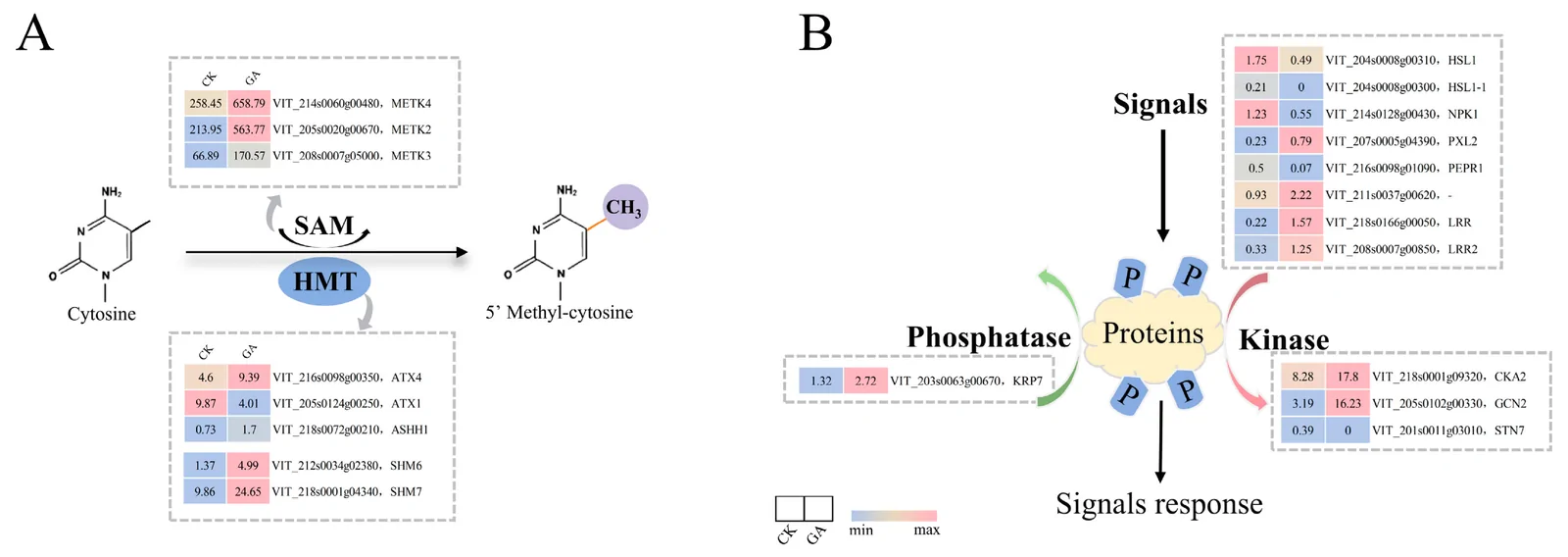
Expression profiles of epigenetic modifications during grape parthenocarpy under GA treatment. (**A**) The expression changes of methylation-related genes. SAM: S-adenosylmethionine; HMT: histone methyltransferase. (**B**) The expression of genes regulating histone phosphorylation.

We identified 20 predicted *VvGAMYB*-associated genes involved in epigenetic modifications in response to GA-induced grape parthenocarpy, including eight genes related to methylation and twelve related to phosphorylation. Regarding methylation-related genes, the expression of S-adenosylmethionine synthetase genes (*VvMETK2/3/4*), which are responsible for producing the methyl donor, was coordinately upregulated by approximately 2.6-fold, a trend opposite to that of *VvGAMYB* (Table S2). Following this, several methyltransferase genes were also upregulated, including serine hydroxy methyltransferase genes (*VvSHM6*, 3.6-fold; *VvSHM7*, 2.5-fold) and histone-lysine N-methyltransferase genes (*VvATX4*, 2.0-fold; *VvASHH1*, 2.3-fold), although *VvATX1* was conversely downregulated by 2.5-fold (Figure 10A). Among the 12 phosphorylation-related regulators, a distinct expression pattern emerged. Kinase genes such as *VvGCN2* (5.1-fold) and *VvCKA2* (2.2-fold) were upregulated, accompanied by four low-expression receptor-like kinases. In contrast, five other receptor-like kinases were downregulated, while the single phosphatase was upregulated by 2.1-fold (Figure 10B). Collectively, these findings suggest that GA may promote grape parthenocarpy through VvGAMYB-mediated reprogramming of epigenetic modifications.

### 2.5. Cis-Element Analysis of Promoters of Selected VvGAMYB-Associated Genes

**Figure 11:**
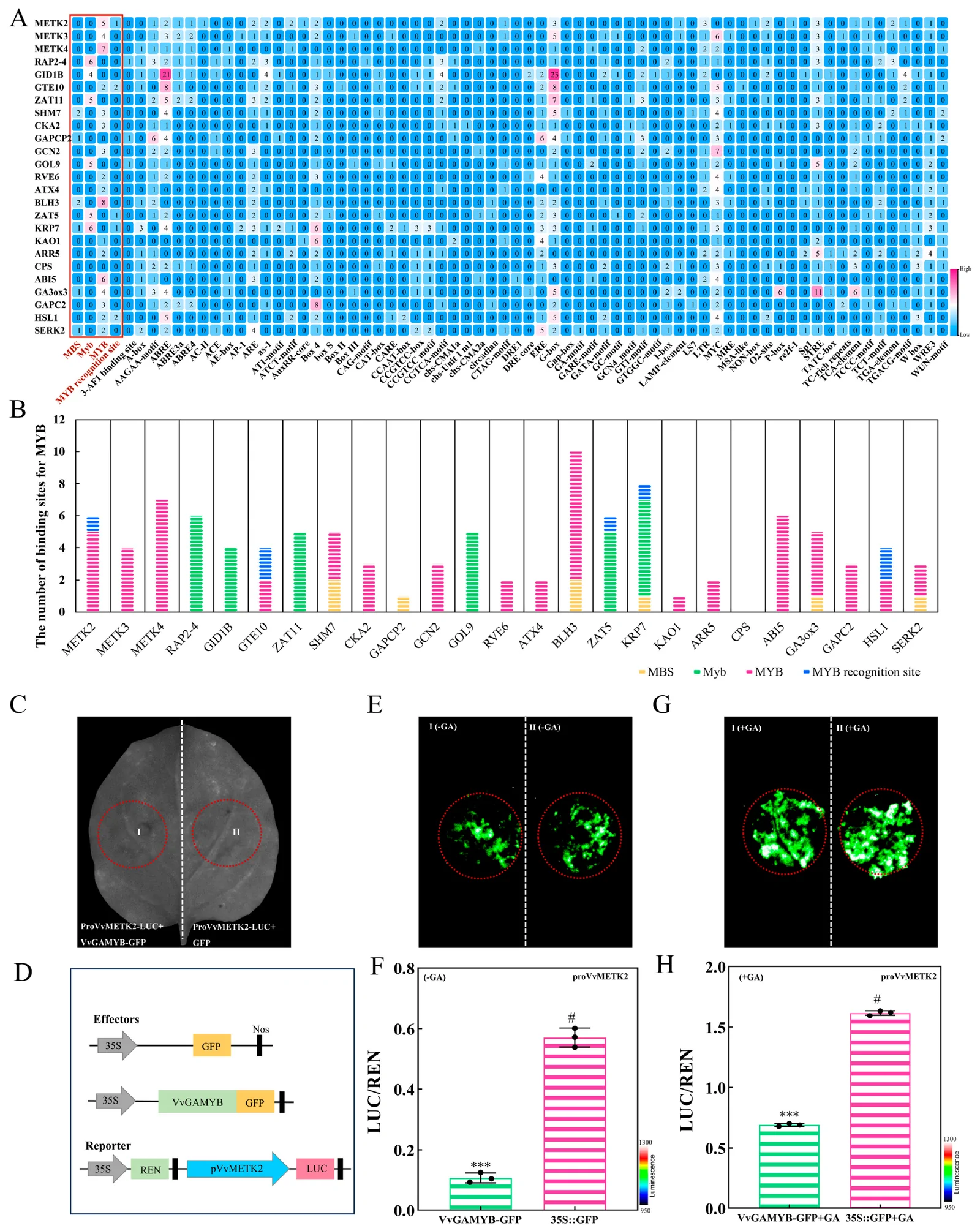
Cis-regulatory element analysis in the promoters and transcriptional regulation validation. (**A**) Heatmap illustrating the distribution and frequency of cis-elements in the promoters of candidate genes. Numbers indicate the actual counts of each cis-element, and the color gradient represents cis-element frequency from low to high. Red highlights MYB-related cis-elements. (**B**) Statistics of the number of MYB transcription factor binding sites in the promoter. Bar chart showing the total number of different types of MYB-related cis-elements (MBS, Myb, MYB, and MYB recognition site) in the promoter regions of each indicated gene. Different colors denote distinct cis-element types. (**C**) Schematic diagram of tobacco leaves used for LUC assays. Red circles indicate the regions used for fluorescence detection. I, Pro*VvMETK2*-LUC+VvGAMYB-GFP; II, Pro*VvMETK2*-LUC+GFP. (**D**) A diagram of vectors used for the LUC assays. 35S, the CaMV 35S promoter; LUC, firefly luciferase; REN, Renilla luciferase. The reporter vector contained *VvMETK2* promoters fused to LUC (empty vector (35S::GFP), VvGAMYB). (**E**) Representative bioluminescence images of tobacco leaves injected with different combinations of vectors, and LUC/REN in tobacco leaves transiently expressing the effectors with reporters containing pro*VvMETK2* under water treatment (−GA). (**F**) Analysis of LUC/REN corresponding to panel (**E**). (**G**) Representative bioluminescence image under GA_3_ treatment (+GA). (**H**) Relative LUC/REN ratios corresponding to panel (**G**). Asterisks indicate significant differences between the indicated groups (*** *p* < 0.001), and # indicates significant differences compared with the corresponding control.

Given that VvGAMYB is a typical R2R3-MYB transcription factor, we next investigated whether selected STRING-predicted VvGAMYB-associated candidates might also be subject to transcriptional regulation by VvGAMYB. We analyzed the cis-acting elements within the 1500 bp promoter regions upstream of the start codon of 25 significantly differentially expressed genes selected from the 181 predicted *VvGAMYB*-associated candidates. Among these, *VvGID1B* contained the largest number of response elements (33), which were primarily light-responsive, whereas *VvKAO1* contained only 12 response elements (Figure 11A). Further analysis revealed that the promoters of all examined genes, except *VvCPS*, contained at least one canonical MYB-binding motif. Notably, the promoter of *VvBLH3* contained 10 such motifs (Figure 11B). The widespread occurrence of MYB-binding motifs suggested that some of these predicted candidates might be transcriptionally regulated by *VvGAMYB*, prompting us to select *VvMETK2* for further experimental validation.

To examine whether VvGAMYB can transcriptionally regulate one of the selected STRING-predicted associated candidates, we performed transient LUC assays in *Nicotiana benthamiana* to test its effect on *VvMETK2*. This gene was significantly upregulated (2.6-fold) by GA treatment and contains MYB-binding motifs in its promoter. The *VvMETK2* promoter was fused to the LUC reporter gene, while *VvGAMYB* driven by the CaMV 35S promoter served as the effector (Figure 11C,D). Co-expression of *VvGAMYB* significantly reduced Pro*VvMETK2*-driven reporter activity compared with the empty-vector control under the no-GA_3_ condition (Figure 11E,F). In contrast, under GA_3_ treatment, the repressive effect of VvGAMYB on Pro*VvMETK2* activity was weakened (Figure 11G,H). These results indicate that VvGAMYB represses *VvMETK2* promoter activity and that GA_3_ treatment partially alleviates this repression. Collectively, this dual regulatory mode may enable VvGAMYB to orchestrate a more precise and amplified signaling cascade during GA-induced parthenocarpy.

## 3. Discussion

Parthenocarpy is a primary determinant of seedlessness in grapes, and GA serves as a key phytohormone for its induction. In practice, exogenous GA application has become the predominant method for producing parthenocarpic seedless berries [43]. In this study, GA treatment not only promoted rapid early ovary development and increased berry length but also repressed ovule growth, ultimately leading to ovule abortion. This dual effect aligns with reports in pear, where GA_4+7_ treatment similarly enhances longitudinal fruit growth [11]. Notably, the most pronounced morphological changes coincided with ovule abortion, consistent with our previous observations [4]. Specifically, browning necrotic spots emerged at the ovule apex by 10 DAF following GA treatment, followed by progressive tissue atrophy and ovule degeneration. These distinct morphological characteristics establish this GA-induced system as an ideal model for dissecting the molecular mechanism underlying GA-mediated seedless berry formation in grape, thereby providing a foundation for identifying key target genes for the molecular breeding of seedless grape varieties.

During GA-induced grape parthenocarpy, numerous genes exhibit strong responses to GA treatment, among which VvGAMYB acts as a key negative regulator [5]. This role is further supported by the identification of several homologous *GAMYB* and *GAMYB-like* genes as GA-responsive factors, which encode a conserved family of R2R3-MYB domain transcription factors localized to the nucleus [44]. Studies on rice anther development suggest that GA activates GAMYB function via the GID1/DELLA perception pathway [35]. In tomato, loss of DELLA function in the *pro* mutant leads to active GA accumulation and induces parthenocarpy [30]. Similarly, in *Arabidopsis*, GA relieves DELLA-mediated repression, promoting miR159 accumulation and subsequently reducing levels of the downstream factor GAMYB [45], an expression pattern consistent with our study. Furthermore, *GAMYB* transcripts are cleaved by miR159 in multiple species, including rice, strawberry, and grape [5,46,47]. In *Arabidopsis* and tomato, miR159 overexpression downregulates miR167 expression by inhibiting *GAMYB*, thereby releasing the silencing of *ARF6/8* and leading to their upregulation. Conversely, miR159 can relieve *GAMYB*-mediated inhibition to upregulate miR167, thereby suppressing *ARF6/8* expression [42,48]. A similar mechanism may have also occurred in our study, as *VvARF6/8* was downregulated in GA-induced grape parthenocarpic berries.

Parthenocarpy relies on the interaction and crosstalk among various hormones [49]. Consistent with this, our findings suggest that VvGAMYB and the STRING-predicted VvGAMYB-associated candidates may regulate grape parthenocarpy through differential expression in response to multiple hormone metabolism and signaling pathways. Exogenous GA application activates the GA biosynthetic pathway, promoting the expression of *VvCPS*, *VvKAO*, and *VvGA3ox3*, while suppressing genes related to *GA2oxs* [50,51]. Moreover, the GA signaling pathway gene *VvGID1B* was downregulated during fruit set, a pattern also observed in tomato and pear [1,11]. GA and CTK often exhibit antagonistic roles across various developmental processes [52,53]. In this study, GA inhibited the expression of the CTK signaling gene *VvARR5*, as well as the ETH signaling genes *VvERF012* and *VvRAP2-4*. Conversely, GA enhanced the expression of the ABA-related gene *VvABI5* and the BR-related gene *VvSERK*, despite the commonly reported antagonism between GA and ABA in other species [11,53,54]. This discrepancy raises the possibility of species-specific differences in hormone crosstalk. However, this interpretation remains a hypothesis that requires further experimental validation, including measurement of endogenous ABA levels. Notably, all the aforementioned genes were identified among the predicted VvGAMYB-associated candidates, implying that exogenous GA may suppress *VvGAMYB* expression and promote parthenocarpy by coordinating a multi-hormone signaling network.

Sucrose, fructose, and glucose are the primary sugars that ultimately enter the fruit for metabolism and accumulation [55]. The positive role of sugars, particularly sucrose and fructose, in promoting parthenocarpic fruit formation has also been demonstrated in cucumber [56]. In this study, sugar levels during fruit set were consistently higher in GA-induced parthenocarpic grape berries compared with those in CK. This may be explained by the downregulation of glycosyltransferase genes involved in sucrose metabolism, which reduces sucrose degradation and thereby promotes sugar accumulation. Given that VvGAMYB acts as a negative regulator in this process, its GA-mediated downregulation may further contribute to these metabolic alterations, ultimately facilitating parthenocarpic berry formation.

Beyond metabolic regulation, our study also uncovered an epigenetic layer involving VvGAMYB. We observed that DNA methylation-related genes identified among the predicted *VvGAMYB*-associated candidates predicted to interact with *VvGAMYB* were upregulated in an expression pattern opposite to that of *VvGAMYB*. This antagonistic relationship suggests that GA may induce parthenocarpy by suppressing *VvGAMYB* expression, thereby relieving its repression on downstream DNA methylation modifiers. This mechanism aligns with a recent finding that *MdMSI1* precisely regulates GA homeostasis and promotes parthenocarpy through H3K27me3 modification [57]. However, an alternative epigenetic pathway has been reported in tomato, where mutation of *MET1* induces parthenocarpy by causing genome-wide methylation dysregulation [58], indicating that GA-independent epigenetic mechanisms also exist across species.

**Figure 12:**
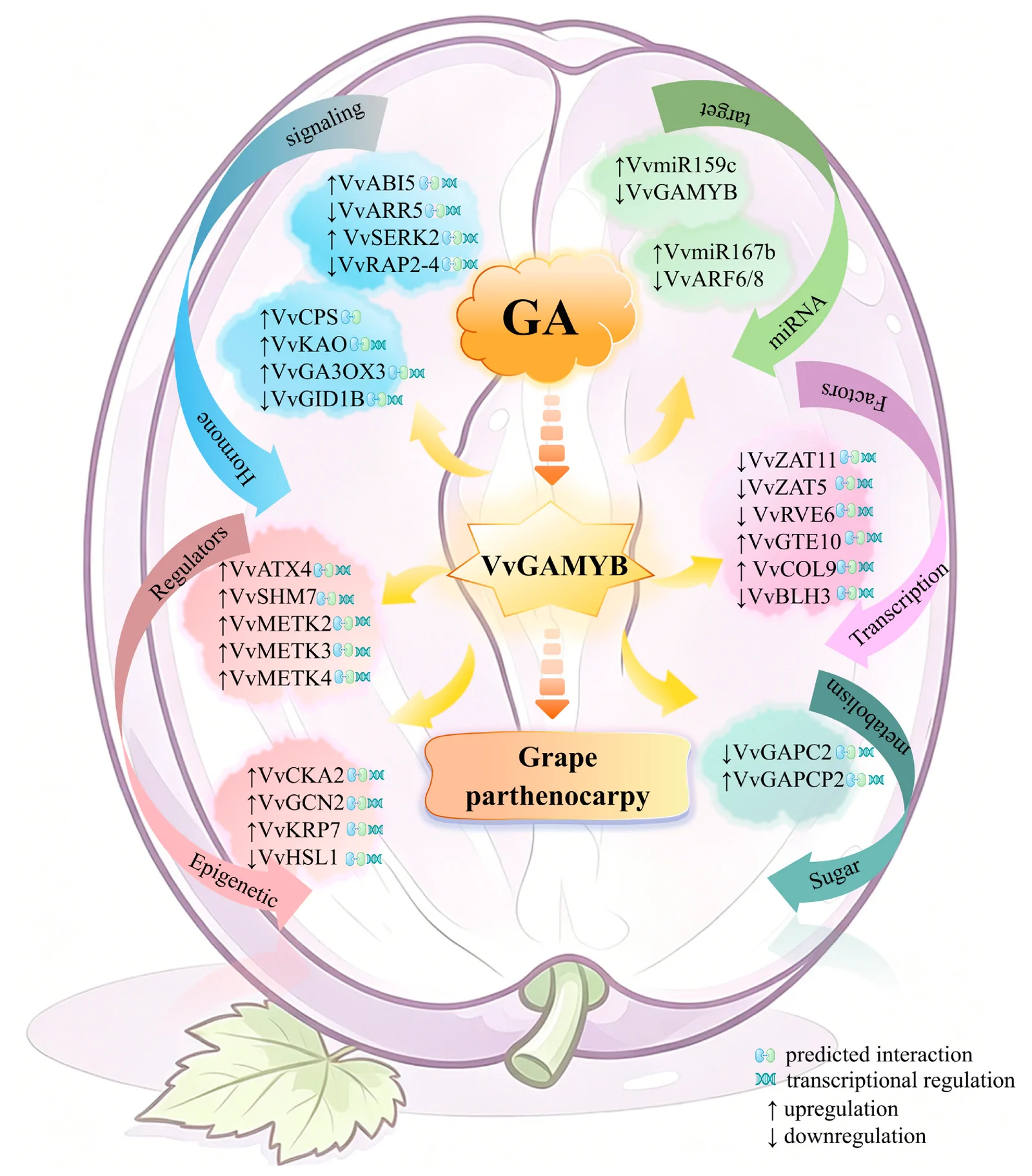
VvGAMYB-associated regulatory network during GA-induced grape parthenocarpy.

To construct a comprehensive regulatory model of GA-induced parthenocarpy, we integrated the identified expression changes into a VvGAMYB-centered multi-signaling network (Figure 12). This network suggests that VvGAMYB might serve as a central hub, integrating multiple hormonal pathways, including GA, ABA, and ethylene signaling. Beyond hormonal crosstalk, VvGAMYB coordinates sucrose metabolic reprogramming and directs a transcriptional cascade involving diverse transcription factors, with epigenetic regulations, such as methylation and phosphorylation modifications, forming an integral part of this framework. Mechanistically, the widespread presence of MYB-binding motifs in the promoters of differentially expressed *VvGAMYB*-interacting genes suggested the potential for direct transcriptional regulation. This hypothesis was experimentally supported by LUC assays, which confirmed that *VvGAMYB* can directly regulate the transcription of selected target genes like *VvMETK2*. Consequently, in the proposed model (Figure 12), we distinguish two layers of regulation: physical protein–protein interactions (predicted) and direct transcriptional regulation (validated), each indicated by specific icons.

## 4. Materials and Methods

### 4.1. Plant Materials and Growth Conditions

The seeded grape cultivar ‘Wink’ was grown under field conditions in Jiangsu Academy of Agricultural Sciences, China. Inflorescence buds were treated 10 days before flowering by dipping them in 50 mg L^−1^ GA_3_ for 30 s, and water was used as the control (CK). The ovaries were field-photographed, stereomicroscopically observed, and measured and sampled at flowering (F) and on different days after flowering (DAF). All tissues were flash-frozen immediately in liquid nitrogen and stored at −80 °C until use.

### 4.2. Determination of Soluble Sugar and Organic Acid Contents

Grape berries collected at different developmental stages were ground into powder in liquid nitrogen. Approximately 0.1–0.2 g of each sample was extracted with 1.5 mL of 80% (*v*/*v*) ethanol at 80 °C for 30 min and centrifuged at 12,000 rpm for 10 min. The pellet was re-extracted with 1.5 mL of 80% ethanol at 80 °C for 10 min and centrifuged again under the same conditions. The supernatants were combined and lyophilized using a vacuum freeze dryer (Labconco, Kansas City, MO, USA), and the dried extracts were redissolved in 10 mL of ultrapure water. A 1 mL aliquot was filtered through a 0.22 μm membrane before high-performance liquid chromatography (HPLC) analysis.

HPLC analysis was performed using an Agilent 1100 series system equipped with a diode-array detector (DAD) and a Discovery C18 column (250 × 4.6 mm, 5 μm) maintained at 30 °C. The mobile phase consisted of methanol and 0.01 mol L^−1^ K_2_HPO_4_ (3:97, *v*/*v*), adjusted to pH 2.4 with phosphoric acid, filtered through a 0.45 μm membrane, and ultrasonically degassed. The flow rate was 0.5 mL min^−1^, the injection volume was 10 μL, and detection was performed at 210 nm. Compounds were identified by comparison of retention times with those of authentic standards and quantified using standard calibration curves. Contents were expressed as mg g^−1^ fresh weight (FW).

### 4.3. Determination of Aspect Ratio

The aspect ratio of the morphology of grape berries and ovules was utilized as a metric, calculated by dividing the longitudinal diameter by the transverse diameter. Measurements of both diameters for individual samples were obtained using Image J2 (v2.14.0). software. The berry aspect ratio was derived as follows: berry longitudinal diameter/berry transverse diameter; the ovule aspect ratio was determined by “ovule longitudinal diameter/ovule transverse diameter”.

### 4.4. RNA Extraction

Total RNA was extracted from different samples of CK and GA-treated grape ovaries during the flowering period (8 May) using a Trizol reagent kit (Invitrogen, Carlsbad, CA, USA) according to the manufacturer’s protocol. RNA quality was assessed on an Agilent 2100 Bioanalyzer (Agilent Technologies, Palo Alto, CA, USA) and checked using RNase-free agarose gel electrophoresis.

### 4.5. RNA Sequencing and Transcriptome Analysis

RNA samples from CK and GA_3_-treated ‘Wink’ grape ovaries collected at flowering were subjected to transcriptome sequencing, with three biological replicates per treatment. After rRNA removal, complementary DNA (cDNA) libraries were constructed and sequenced on the Illumina HiSeq 4000 (Illumina, Inc., San Diego, CA, USA) platform. Raw reads were quality-filtered using fastp (v0.18.0), and residual rRNA reads were removed using Bowtie2 (v2.5.2). The remaining clean reads were aligned to the *Vitis vinifera* reference genome using HISAT2 (v2.1.0). Transcripts were reconstructed using String Tie (v1.3.4), and gene expression levels were quantified using RSEM (v1.2.19). Differential expression analysis was performed using DESeq2 (v1.36.6) based on read-count data. Genes with |log_2_FC| > 1 and FDR < 0.05 were considered differentially expressed genes (DEGs). RNA-seq quality metrics and the complete lists of identified genes and DEGs are provided in Supplementary Table S3.

### 4.6. Vector Construction

cDNA was synthesized by reverse transcription of total RNA with the Hifair^®^ AdvanceFast 1st Strand cDNA Synthesis Kit (Yeasen Biotech Co., Ltd., Shanghai, China) and was subsequently used for amplification or expression analysis.

Polymerase chain reaction (PCR) was performed using DNA or cDNA as the template with gene-specific primers. Amplification was carried out with the 2× Hieff™ PCR Master Mix (Yeasen Biotech) according to the manufacturer’s recommended reaction system and thermal cycling conditions, with primers listed in Table S1. The PCR products were verified by agarose gel electrophoresis and purified using the FastPure Gel DNA Extraction Mini Kit (Vazyme Biotech Co., Ltd., Nanjing, China) following the manufacturer’s instructions.

The ClonExpress^®^II One Step Cloning Kit (Vazyme Biotech) was used to recombine the target fragment into the corresponding vector, which was cut by the corresponding restriction enzymes in advance, thus obtaining the corresponding recombinant plasmids.

### 4.7. Reverse Transcription Quantitative PCR

RT-qPCR was performed using Hieff^®^ qPCR SYBR Green Master Mix (Yeasen Biotech) with primers listed in Table S1. Raw fluorescence data acquired from the My-IQ 2 detection system were exported to Microsoft Excel for analysis. And the relative expression levels of target genes were calculated using the 2^−ΔΔCt^ method [59].

### 4.8. Subcellular Localization

Leaves of five-to-six-week-old *Nicotiana benthamiana* L. plants were used as experimental materials. *Agrobacterium tumefaciens* harboring the pSuper1300-VvGAMYB construct was cultured to an optical density at 600 nm (OD_600_) of 1.0, harvested by centrifugation at 5000 rpm for 10 min, and washed with sterile water. The pellet was resuspended in inoculation buffer (10 mM 2-(N-morpholino)ethanesulfonic acid (MES), 10 mM MgCl_2_, 0.15 mM AS, 2% sucrose) and incubated for 3 h. *N. benthamiana* leaves were infiltrated with *Agrobacterium* carrying the pSuper1300-VvGAMYB vector. After infiltration, the leaves were kept at 25 °C in darkness for three days. DAPI Stain Solution (Yeasen Biotech) was used for nuclear staining, and GFP and 4′,6-diamidino-2-phenylindole (DAPI) fluorescence signals were observed with a confocal microscope (ZEISS, LSM800, Oberkochen, Germany).

### 4.9. Dual-Luciferase Assay

The ORF of *VvGAMYB* and the 1–1500 bp upstream promoter regions of *VvMETK2* were inserted into the pSuper1300 and pGreenII0800-LUC vectors to generate the effector pSuper1300-VvGAMYB and reporter pGreenII0800-LUC-VvMETK2, respectively. The recombinant constructs were introduced into *N. benthamiana* leaves via *Agrobacterium* GV3101 (pSoup-p19) infiltration, and luminescence signals were captured using a Tanon (Tanon Science & Technology Co., Ltd., Shanghai, China) chemiluminescence imaging system. Firefly luciferase (LUC) and Renilla luciferase (REN) activities were measured using the Dual Luciferase Reporter Gene Assay Kit (Yeasen Biotech) according to the manufacturer’s instructions.

### 4.10. Bioinformatics Analysis

Theoretical gene and protein sequences of grape species were obtained from the CRIBI database. The hmmscan online program (https://www.ebi.ac.uk/Tools/hmmer/search/hmmscan, accessed on 8 June 2024) was used to do the domain analysis, and then the Ensembl Plants database (http://plants.ensembl.org/, accessed on 15 June 2024) was used to screen the genes containing specific domains. The protein structure analysis was performed on the COBALT website (https://www.ncbi.nlm.nih.gov/tools/cobalt/cobalt.cgi?CMD=Web, accessed on 24 June 2024).

Heatmaps were generated using TBtools-II (v2.390). For RNA-seq expression heatmaps, the numerical values shown in the cells represent the mean FPKM values of three biological replicates. Colors were directly mapped to the corresponding mean FPKM values from low to high. For the RT-qPCR heatmap of *VvGAMYB*, relative expression was calculated using the 2^−ΔΔCt^ method. For the cis-element heatmap, the numerical values and color scale represent the actual numbers of cis-elements.

The nucleotide and amino acid sequences of *VvGAMYB* were analyzed by DNANMAN6 (v6.0.3) software, and its physical and chemical parameters were all shown in the Expasy program (https://web.expasy.org/protparam/, accessed on 5 July 2024). The prediction of transmembrane helices, hydrophilicity and the signal peptide condition was performed by TMHMM-2.0 (http://www.cbs.dtu.dk/services/TMHMM/, accessed on 15 July 2024), ProtScale (http://web.expasy.org/protscale/, accessed on 20 July 2024), and SignalP-5.0 (https://services.healthtech.dtu.dk/service.php?SignalP-5.0, accessed on 5 August 2024), respectively.

The corresponding GAMYB sequences of 24 different species were obtained by National Center for Biotechnology Information (NCBI) BLAST (https://blast.ncbi.nlm.nih.gov/Blast.cgi, accessed on 9 August 2024). These protein sequences include AtMYB33 (NP_001078537.1), NtGAMYB (XP_016484620.1), FvGAMYB (*Fragaria vesca*, XP_011468770.1), SlGAMYB2 (XP_010322306.1), ZjMYB33 (*Ziziphus jujuba*, XP_015876153.1), PpGAMYB (XP_007218962.1), PtMYB20 (*Populus trichocarpa*, XP_006372565.2), PmGAMYB (*Prunus mume*, XP_008231582.1), MdGAMYB (XP_008346342.2), PbGAMYB (*Pyrus* × *bretschneideri*, XP_009355091.1), CcMYB33 (*Citrus clementina*, XP_006432323.2), DkMYB8 (*Diospyros kaki*, AKP06176.1), PgGAMYB (*Punica granatum*, XP_031377075.1), PsMYB10 (*Prunus salicina*, ABX79946.1), EjMYB8 (*Eriobotrya japonica*, AOQ26228.1), LcMYB1 (*Litchi chinensis*, APP94121.1), MaGAMYB (*Musa acuminata*, XP_009398961.1), OsGAMYB (XP_015622335.1), ZmGAMYB (*Zea mays*, NP_001130632.1), GmGAMYB (*Glycine max*, NP_001304541.1), CaGAMYB (*Capsicum annuum*, XP_016578021.1), CsMYB33 (*Cucumis sativus*, XP_004140923.1), and AcGAMYB (*Ananas comosus*, XP_020113516.1). Phylogenetic tree construction was performed with MEGA 5.1 software (accessed on 10 August 2024) through the neighbor-joining method. MEME online software (http://meme-suite.org/tools/meme, accessed on 17 August 2024) was used for the analysis of sequence structure and conserved sequences.

The online software STRING (http://string-db.org/, accessed on 27 August 2024) was used to predict the proteins associated with VvGAMYB. KEGG (https://www.kegg.jp/kegg/pathway.html, accessed on 29 August 2024) and Gene Ontology (GO) (http://geneontology.org/, accessed on 30 August 2024) analyses were performed to unscramble the interaction network of VvGAMYB.

### 4.11. Statistical Analysis

All experiments were performed with three biological replicates. For non-transcriptomic experimental comparisons, data are presented as mean ± standard deviation (SD), and statistical significance was evaluated using Student’s *t*-test, with *p* < 0.05 considered statistically significant. Differential expression analysis of the RNA-seq data was performed using DESeq2 as described above.

## 5. Conclusions

In conclusion, GA-induced seedlessness in grape arises from a highly complex regulatory network that integrates hormone crosstalk (GA, ABA, ethylene, CTK, and BR), sugar metabolism, and epigenetic modifications, with VvGAMYB functioning as a central regulatory hub. Notably, this regulatory system exhibits diversity across different species and even among different seedless types within the same species. Collectively, our findings establish a theoretical foundation for further elucidating the regulatory mechanisms underlying GA-mediated seedless traits and the downstream pathways orchestrated by VvGAMYB.

## Data Availability

The raw RNA-seq data generated in this study have been deposited in the NCBI Sequence Read Archive (SRA) under accession number PRJNA1533705.

## Supplementary Materials

The following supporting information can be downloaded at https://www.mdpi.com/article/10.3390/ijms27198828/s1.
