# Supplementary material for: VvGAMYB Orchestrates Multi-Signaling Networks to Modulate GA-Induced Parthenocarpic Seedless Berry Development in Grapes
Source: Int J Mol Sci. 2026 Oct 2;27(19):8828. doi: 10.3390/ijms27198828 (PMC13665188; doi:10.3390/ijms27198828)
Supplement: Supplementary file 1 [file ijms-27-08828-s001.zip › Supplementary material.pdf]

VvGAMYB orchestrates multi-signaling networks to modulate GA-induced parthenocarpic seedless berry development in grapes

Qiqi Wu[1#], Ruiqiang Chao[1#], Linjia Luo[1], Yuqing Huang[1], Jing Li[1], Lianshuang Liang[1], Xuxian Xuan[1], Yanan Liu[1], Xinpeng Chen[1], Pengcheng Zhao[2], Xicheng Wang[3], Chen Wang[1*]

1 College of Horticulture, Nanjing Agricultural University, Nanjing 211800, China

2 College of Agronomy and Horticulture, Jiangsu Vocational College of Agriculture and Forestry, Jurong 212400, China

3 Institute of Pomology, Jiangsu Academy of Agricultural Sciences, Nanjing 210014, China

* Corresponding Author:

Chen Wang: wangchen@njau.edu.cn

# These authors contributed equally to this work.

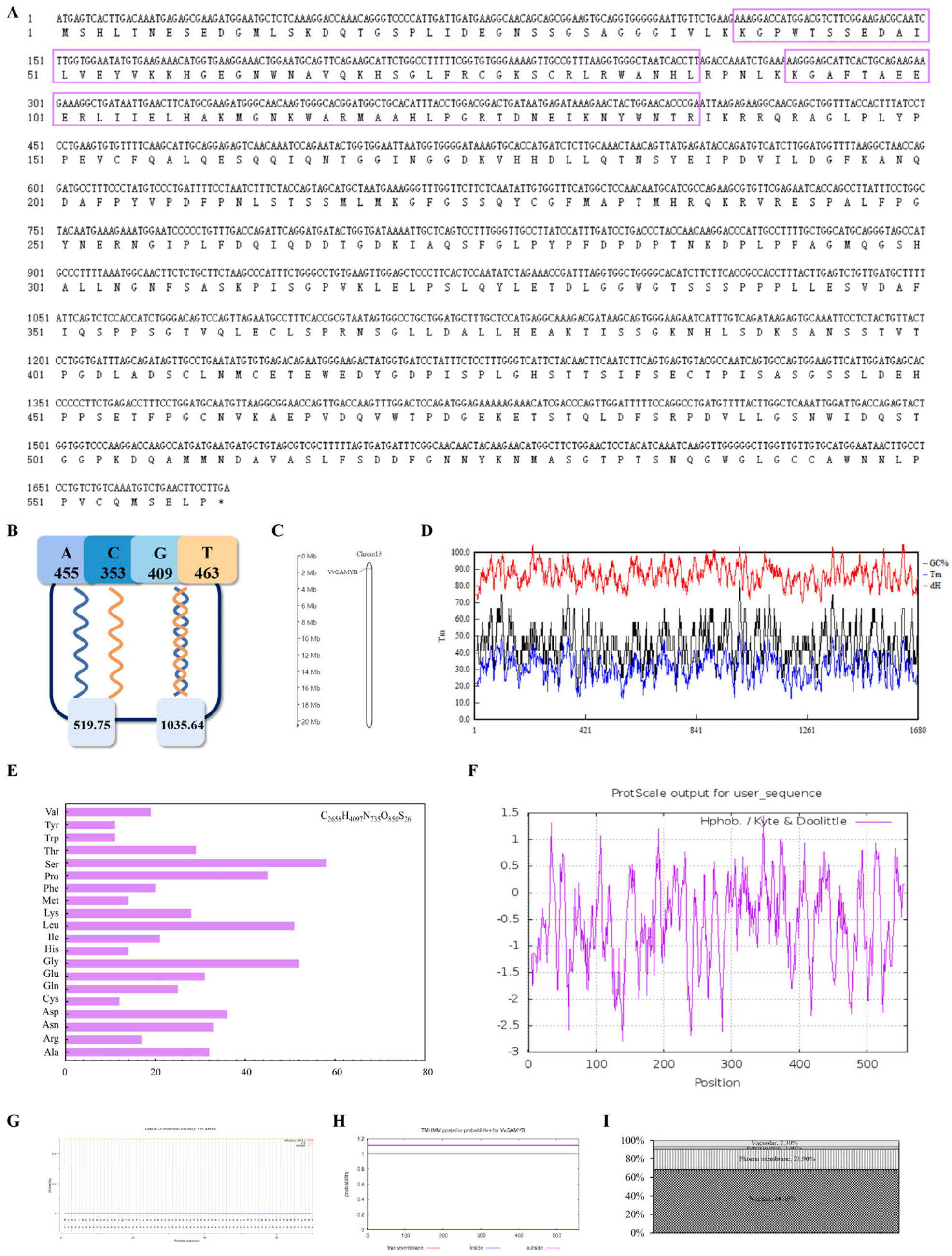

Figure S1 Sequence and bioinformatic characterization of VvGAMYB. (**A**) Nucleotide sequence and deduced amino acid sequence of VvGAMYB. (**B**) Nucleotide composition of VvGAMYB. (**C**) Chromosomal location of VvGAMYB on chromosome 13. (**D**) Profiles of G+C content, melting temperature (Tm), and enthalpy (dH) along the VvGAMYB nucleotide sequence. (**E**) Amino acid composition of VvGAMYB. (**F**) Hydropathy profile of VvGAMYB. (**G**) Signal peptide prediction of VvGAMYB. (**H**) Transmembrane helix prediction of VvGAMYB. (**I**) Subcellular localization prediction of VvGAMYB.

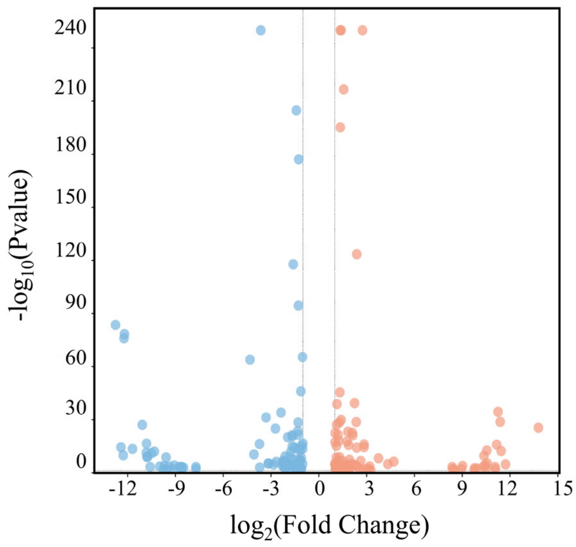

Figure S2 Volcano plot of differentially expressed genes among the predicted VvGAMYB-associated candidates. Orange dots indicate significantly up-regulated genes ($log_2FC > 1$ and $FDR < 0.05$), whereas blue dots indicate significantly down-regulated genes ($log_2FC < -1$ and $FDR < 0.05$). log_2FC, log_2 fold-change; FDR: false discovery rate.

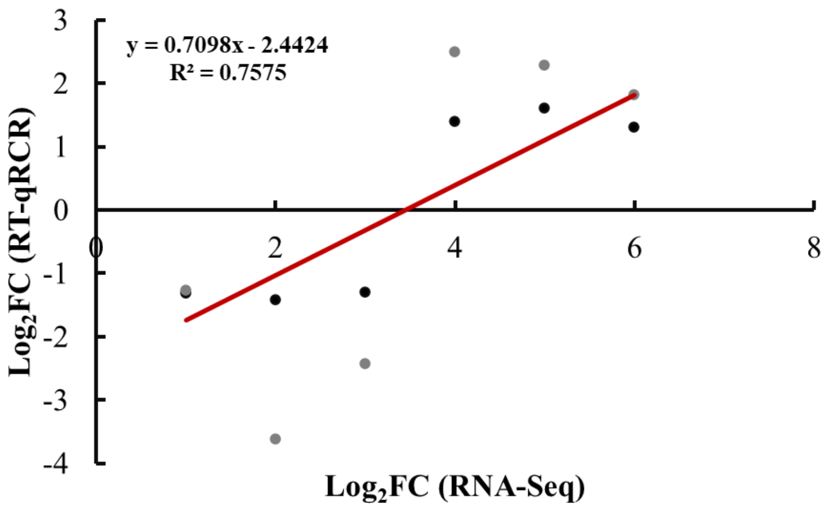

Figure S3 Correlation between RNA-seq and RT-qPCR-derived fold changes for selected genes. The X and Y axes represent the log_2 fold changes calculated from the RT-qPCR and RNA sequencing data, respectively. The R-squared values indicate the strength of the relationship and the goodness of fit for the linear regressions.
